# Oral toxicity produced by chemotherapy: A systematic review

**DOI:** 10.4317/jced.51337

**Published:** 2014-02-01

**Authors:** Begonya Chaveli-López

**Affiliations:** 1 Valencia University Medical and Dental School, Valencia, Spain

## Abstract

Introduction: Antineoplastic chemotherapy remains one of the most widely used management strategies in cancer, either alone or in combination with other types of treatment. The main inconvenience of chemotherapy is its lack of selectivity, since it acts upon both tumor cells and rapidly multiplying normal cells such as bone marrow cells, hair follicle cells and oral and gastrointestinal mucosal cells. Material and method: An exhaustive search was made of the main oral toxic effects of chemotherapy in the PubMed-Medline, Cochrane Library and Scopus databases. A total of 1293 articles were identified, of which 333 met the study inclusion criteria. Results: The toxic effects of chemotherapy at oral mucosal level comprise mucositis, osteonecrosis of the jaws secondary to bisphosphonate use, susceptibility to infections, dental alterations, salivary and neurological disorders, dysgeusia and bleeding tendency. These complications have a negative impact upon patient quality of life, and in some cases can prove life-threatening. Conclusions: Evaluation of patient oral and dental health is essential before administering chemotherapy, in order to minimize the risk of oral and systemic complications of such treatment.

** Key words:**Chemotherapy, oral complications, dental, saliva and osteonecrosis jaw.

## Introduction

In global terms, cancer is characterized by increased cell proliferation and diminished apoptosis (1). The proliferation of atypical cells gives rise to invasive capacity, with the infiltration of body tissues or organs through the bloodstream or lymphatic system – this process being known as metastasis. The existing cancer treatments comprise surgery and radiotherapy, chemotherapy, biological or immune therapy, hormonal therapy and gene thera-py (a form of treatment that is still in the investigational stage) (2), which aim to block cell proliferation (1). Despite the advances in cancer management, chemotherapy remains one of the most widely used treatment modalities, either alone or in combination with other types of treatment (3,4). The great inconvenience of chemotherapy is its lack of selectivity, since it acts upon both tumor cells and rapidly multiplying normal cells (1,3-5).

The oral cavity is very susceptible to the direct and indirect toxic effects of chemotherapy. This is due to a number of factors, including the high cellular turnover rate of the oral mucosa, the complex and diverse microflora of the oral cavity, and oral tissue trauma occurring during normal oral function (3,4). It is therefore essential to evaluate the oral condition of the patient and to stabilize any oral disease conditions before cancer treatment is provided (5). Oncological patient care must be viewed from both the preventive and therapeutic perspectives, in order to minimize the risk of oral complications and other related systemic complications (4).

The present systematic review offers an update on the main oral toxic effects of chemotherapy, based on the data found in the scientific literature.

## Material and Methods

An exhaustive search was made of the PubMed-Medline, Cochrane Library and Scopus databases, using the following keywords: *“oral complications”, “oral mucositis”, “oral candidiasis”, “periodontal disease”, “gin-givitis”, “caries”, “oral infection”, “dental development”, “dysgeusia”, “taste disturbances”, “saliva”* and *“osteonecrosis jaw”*, related by means of the boolean operators “AND” and “NOT” to the terms *“chemotherapy”* and *“radiotherapy”*, respectively. We included human studies as well as reviews published in English or Spanish during the last 10 years (from January 2002 to December 2012). Opinion articles, series with fewer than 5 cases, and studies involving radiotherapy and/or the bone marrow transplantation as sole or concomitant treatments were excluded. A total of 1293 articles were identified, of which 333 met the study inclusion criteria ( Table 1).

**Table 1:**
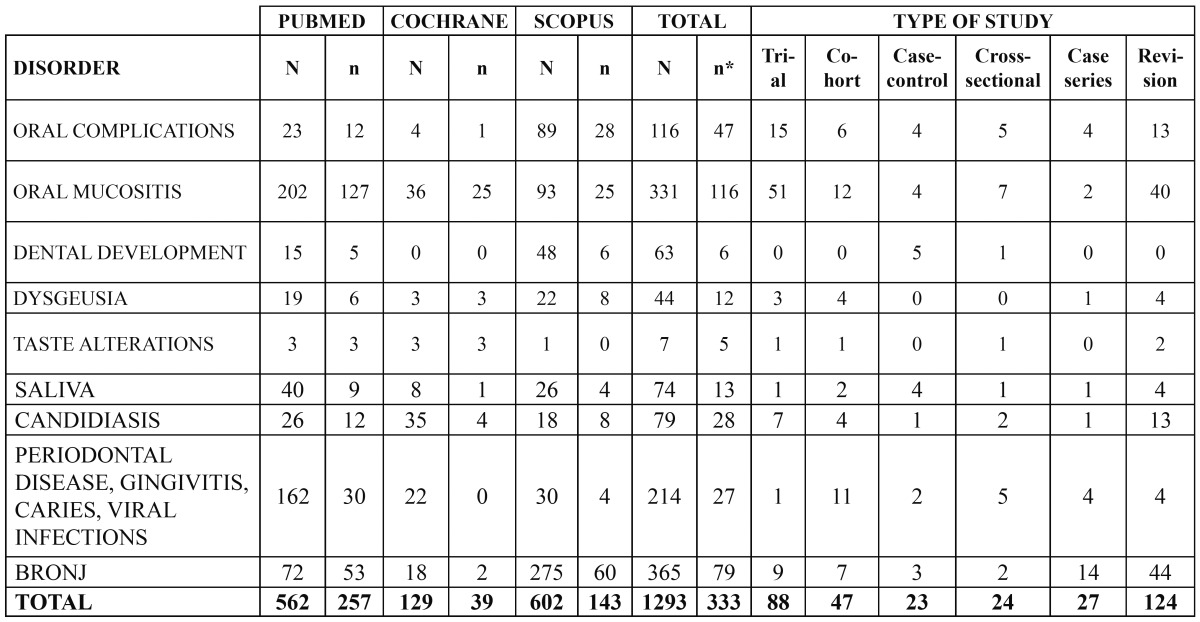
Types of reviewed studies on the oral complications of chemotherapy published in the literature (2002-2012). // N: studies identified from the search; n: selected studies; n*: selected studies eliminating articles appearing in more than one database; BRONJ: bisphosphonate-related osteonecrosis of the jaw

## Oral toxicity produced by the administration of chemotherapy

The oral complications of chemotherapy are either a result of direct action of the drug upon the oral mucosa (direct stomatological toxicity), or an indirect consequence of chemotherapeutic drug-induced bone marrow suppression or myelosuppression (indirect stomatological toxicity)(3,4).

1.Mucosal toxicity

The cells of the oral cavity have a fast turnover rate, with a cycle of 7-14 days. This explains the special susceptibility of the oral mucosa to the toxic effects of cytostatic drugs (3).

-Terminology

There is some debate regarding the terminology used in reference to the mucosal alterations produced by cancer treatment. Some authors prefer the term “stomatitis”, since the term “mucositis” can refer to any mucosal membrane of the gastrointestinal tract (6). However, there appears to be agreement in the international literature on the use of the term “oral mucositis” or “buccal mucositis”. This term, introduced in the late 1980s, refers to inflammation of the oral mucosa induced by radiotherapy (seen in 80% of all patients), chemotherapy (in approximately 40-50%), or bone marrow transplantation (in over 75% of all patients), and is considered a manifestation of leukopenia (4,6).

-Physiopathology

The pathogenesis of oral mucositis has not been fully clarified. According to the hypothesis of Sonis et al., mucositis comprises four phases. The first phase (inflammatory/vascular phase) occurs after the administration of chemotherapy, with the release of cytokines from the epithelium (tumor necrosis factor-alpha, interleukins 1 and 6), producing local tissue damage that leads to early stage mucositis. In phase 2, and as a result of the cancer treatment, epithelial renewal or turnover decreases, with mucosal atrophy and ulceration. These first two phases manifest about 0-5 days after treatment administration. Phase 3 develops one week after the start of antineoplastic therapy, and is characterized by epithelial rupture and the appearance of a fibrinous exudate that favors the development of pseudomembranes and ulcers. This is generally the most symptomatic phase, since it coincides with the period of maximum neutropenia or secondary bacterial colonization (1). The fourth phase involves healing (cicatrization) and generally occurs after 12-16 days. It is dependent upon the proliferative capacity of the epithelium, hematopoietic recovery, restoration of the oral microflora, and the absence of factors such as infection and mechanical irritation (7).

-Risk factors

A number of risk factors influencing the frequency and severity of mucositis have been described. Some are inherent to the patient, such as the type of tumor (hematological disease)(6,8), age (young patients)(6), oral and dental health (poor oral hygiene before and during chemotherapy)(9), the nutritional condition of the patient, and the maintenance of liver and kidney function. In turn, other risk factors are related to the administered drug, such as the type of cytostatic agent used ( Table 2)(10), the frequency of administration (prolonged or repeated low-dose administration), and concomitant treatment in the form of radiotherapy and/or bone marrow transplantation (1).

**Table 2:**
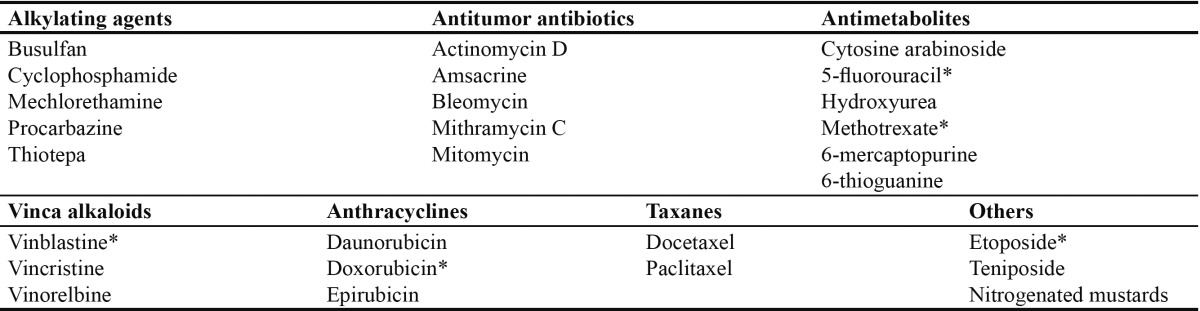
Principal chemotherapeutic drugs capable of causing oral mucosal lesions. *cytostatic drugs that most often produce mucositis.

-Clinical characteristics

Mucositis manifests as reddening (erythema), edema or ulceration that can be accompanied by a mild burning sensation. Extreme presentations in turn are characterized by large and painful ulcers that have a strong impact upon patient quality of life – limiting basic functions such as speech, eating or the swallowing saliva (2-4). These manifestations appear shortly after the start of treatment. In 18-40% of all cases they develop after administration of the first chemotherapy cycle (6). Peak symptoms expression is reached after one week, followed by gradual resolution within 2-3 weeks after the end of chemotherapy, provided there is no bone marrow suppression. Mucositis can often become overinfected, mainly with herpes simplex virus or Candida albicans, particularly in patients with prolonged neutropenia.

-Evaluation

A number of systems have been developed for measurement and quantification of the oral epithelial changes, including general scales, multiple variable scores, and treatment-specific classifications (6). At present, the general scale of the World Health Organization (WHO) is the most widely used system in research, combining the clinical appearance of the mucosa with the capacity of the patient to swallow food (4-6,8). In contrast, in the clinical setting, the most commonly used system is the treatment-specific classification pertaining to the Common Toxicity Criteria for Adverse Events of the United States National Cancer Institute (NCI), which encompasses the patient symptoms, the capacity to swallow food, and the need for treatments (1).

-Diagnosis

The diagnosis of mucositis is established from the clinical characteristics. As complementary tests, studies can be made of lesion samples when overinfection due to Candida, herpes simplex virus or bacteria is suspected. In addition, a blood test can prove useful, since neutropenia, thrombopenia and dehydration are often associated. The differential diagnosis includes viral, bacterial and fungal oropharyngeal infections, and graft-versus-host disease. These are disorders requiring careful consideration, and which require specific and timely management (1).

-Treatment and prevention

Correct oral hygiene and a good gingival condition during chemotherapy are associated to a lesser incidence and severity of mucositis (4,9). Regarding the use of drugs or substances for the prevention and treatment of mucositis, the literature offers contradictory information (1,3,8,9). Good results have been reported with the application of ice before and during chemotherapy (1,4,8), and also with the use of iseganan hydrochloride (11). However, the systematic review published by Worthington et al. (12) found that a number of the treatments used for the prevention of mucositis (amifostine, benzidamine, calcium phosphate, Chinese traditional medicine, etoposide in bolus form, honey, hydrolytic enzymes, pieces of ice, iseganan, oral care and zinc sulfate) offer only limited benefit, and their application moreover depends upon the characteristics of the patient. Other treatment such as palifermin (13), oral glutamine (14), granulocyte colony stimulating factor (G-CSF) and macrophages in rinses, the topical application of polyvinylpyrrolidone (PVP) and hyaluronic acid (15), and low-intensity laser photot-herapy, have been related to a decrease in the appearance and severity of mucositis (1). Although no treatment has been shown to successfully eliminate mucositis, management of the pain symptoms with anesthetic solutions and, according to recent studies, with morphine in the form of rinses (1,4,8), can help lessen oral discomfort and improve patient quality of life. The use of artificial saliva or cholinergic agonists, and the intake of abundant liquids for the prevention of hyposalivation, help preserve the integrity of the oral mucosa (8). On the other hand, alcohol and smoking should be avoided. Antifungals in suspension or as pomades, either isolatedly or in combination with chlorhexidine, are used for the prevention and treatment of overinfection with Candida (16).

2.Dental alterations

Chemotherapy can cause a range of aesthetic and functional dental problems, mostly in children treated before 5 years of age. However, prepubertal children are also at risk of suffering such late effects (17).

- Physiopathology

In contrast to radiotherapy, which only affects the cells of the irradiated zone, chemotherapy exerts a systemic effect. Due to the short half-life of cytostatic drugs, the dental defects are generally localized, and are secondary to transient changes in odontoblast function, rather than apoptosis (4,17).

•Crown and root morphology

The shape and size of the crown in the temporal dentition are not affected, since crown morphology is determined before birth. However, in the case of the permanent dentition, we can observe macrodontia with a prevalence of 2.2-5.2%, due to the action of certain chemotherapeutic drugs such as vinblastine and vincristine upon the mature odontoblasts and ameloblasts (17). Chemotherapy also causes morphological anomalies of the dental roots. In this context, in children under 5 years of age we can observe alterations of the roots of the upper and lower premolars, while older children show alterations of the roots of the upper and lower molars, premolars and canines (17). The action of cytostatic drugs upon the microtubules of the odontoblasts interrupts the formation of collagen fibrils and dentinal matrix secretion, giving rise to thin and sharp-pointed roots.

•Agenesis-hypodontia

Intensive chemotherapy, or chemotherapy involving several treatment cycles in the initial stages of hard tissue formation, can give rise to dental agenesis (17,18).

•Dental hypoplasia

Hypoplasia is characterized by small grooves, point defects and fissures in the enamel in mild cases, horizontal rows of deep grooves in severe cases, and the absence of dental enamel in extreme cases (17). Vincristine, vinblastine and cyclophosphamide are the drugs most commonly related to the appearance of hypoplasias, discolorations and opacification of the enamel, due to their action upon odontogenesis (18).

•Caries

Some authors have described an increased incidence of caries in children subjected to chemotherapy, though the data are controversial, since caries may result from an increased use of rinses, often with a high sugar content, to treat hyposalivation (17,18). In adults, a number of studies have reported an increase in caries in patients subjected to chemotherapy (3,7).

-Treatment

Although the effects of cancer treatment upon the oral cavity are inevitable, a series of measures should be adopted to ensure that their impact upon patient quality of life is minimized. Children scheduled for chemotherapy should undergo a thorough clinical and radiological evaluation by the dentist (4,17). Periodic checks should be made, every 6 months, with tartrectomy and the application of fluor in the dental office. The recommended tooth brushing frequency varies, though at least two daily brushings are advised, using fluorated toothpaste (17). Chlorhexidine varnish also can be applied twice a day as a preventive measure against caries (10,17).

3.Neurological disorders

-Etiology

Certain types of chemotherapeutic drugs, such as vincristine and vinblastine, can exert direct neurotoxic effects (4).

-Clinical characteristics

Neurotoxicity accounts for 6% of all oral complications, causing discomfort and pain similar to that of pulpitis. The pain sensation is constant and of sudden onset, affecting the region of the lower molars in the absence of dental disease.

-Treatment

An oral and radiological exploration should be made to distinguish the pain from that of pulp origin. The symptoms usually disappear one week after chemotherapy. In some cases dental hypersensitivity can manifest weeks or months later. In these cases topical fluoride or the use of a desensitizing toothpaste may lessen the symptoms (4).

4.Salivary alterations

Saliva plays an important role in the modulation of oral health. In this context, deficits in the amount and quality of the gland secretions can exert negative effects upon oral mucosal health (17).

•Salivary immunoglobulins

Chemotherapy has been shown to affect a series of salivary components, such as immunoglobulins, peroxidases, amylases and other proteins. Salivary immunoglobulins protect the mucosa against traumatisms and microbial infections. A decrease in the salivary production of immunoglobulins IgG and IgA could explain some of the oral complications of chemotherapy. In this sense, a decrease in IgA has been associated to the appearance of mucositis in patients receiving chemotherapy (10). Jankovic et al., in a study of the effects of cytostatic drugs such as anthracycline and 5-FU in 40 patients with different metastatic tumors, recorded lower IgA levels and higher IgG concentrations in unstimulated saliva versus the control group, with an IgG/IgA ratio of 1.53 in patients with mucositis, while the healthy subjects presented values of under 1.0 (10).

•Salivary pH and chemotherapy

Some authors have reported a modification in salivary buffer capacity after the administration of chemotherapy (19). However, other investigators such as Avşar et al. (17) or Rojas-Morales et al. (19) have observed no significant variations following the administration of cytostatic agents.

•Xerostomia

Chemotherapy can give rise to a temporary but clinically significant decrease in salivary flow that improves as the bone marrow recovers (4,17). Such a decrease in salivary flow in turn favors the appearance of mucositis (1,6-9). The symptoms of xerostomia or dry mouth include dryness, burning sensation or discomfort (particularly of the tongue), cracked lips, changes in the tongue surface, and problems in wearing removable dentures or drinking liquids. The condition tends to be preceded by a metallic taste sensation that subsequently can lead to dysgeusia and glossodynia secondary to the effects of chemotherapy upon the tongue papillae and demineralization of the nerve fibers (7,10).

In treating xerostomia it is advisable to maintain adequate oral hydration by means of the regular intake of water, the use of saliva substitutes or cholinergic agonists such as pilocarpine, cevimeline or bethanechol (when pilocarpine proves ineffective); these measures moreover favor integrity of the oral mucosa (8,17).

5.Dysgeusia

According to some estimates, 50-75% of all cancer patients who receive chemotherapy, radiotherapy or both can experience alterations in taste perception (8).

-Etiopathogenesis

The main cause of dysgeusia in cancer patients is the action of chemotherapy and radiotherapy upon oral epithelial cell turnover, and the effects of such treatments upon nerves, taste buds and olfactory receptors (4,10). On the other hand, anticancer drugs can access the oral cavity through diffusion from plasma in the capillaries, producing an unpleasant taste (8,20). The mechanisms underlying dysgeusia also may be related to modifications in the concentrations of sodium, potassium and calcium in the taste bud cell receptors (20). Other causes are candidiasis, viral infections and gingivitis, among others.

-Clinical characteristics

The patients present distorted taste sensation, describing a metallic or very salty taste of food. These situations can adversely affect patient food intake and nutritional condition.

-Measurement of taste sensitivity

The evaluation of patients with taste alterations requires a good case history, together with specific questioning (8). We can also deposit solutions with the primary flavors on the back of the tongue, with the purpose of determining whether the patient is able to correctly identify the flavors. Another much less frequently used test is electric stimulation (galvanometry), delivering an electric current of several microamperes onto the back of the tongue, to assess patient capacity to identify the stimulus.

-Treatment

Although dysgeusia has multiple origins, there are simple forms of treatment, such as a reduction of the dose of certain chemotherapeutic drugs (e.g., histone deacetylase inhibitors), the treatment of oral infections, and dietetic counseling (8,20). In relation to this latter aspect, it is advisable to increase liquid intake with meals, and chew food slowly - thereby freeing more flavors and especially increasing saliva production. In addition, diversity during meals is advisable, in order to prevent taste bud adaptation to flavors. Other pharmacological strategies include zinc supplements and amifostine. However, the results obtained in different clinical trials have not been entirely satisfactory, and other treatment alternatives, such as vitamin D supplements, are therefore being investigated (8).

6.Infections

Cytostatic agents can affect the bone marrow, producing anemia, leukopenia and thrombopenia. As a result of their indirect toxicity mechanism, the oral cavity becomes more vulnerable to infections approximately one week following the administration of these drugs. Bone marrow function must be evaluated, since the reduction or absence of inflammatory phenomena causes the oral tissues to appear normal; infections therefore go unnoticed, and septicemia may result. It should be noted that apart from causing frequent infections, agranulocytosis also produces neutropenic ulcers, which are characterized by a central necrotic area, no perilesional erythematous halo, and irregular margins. These ulcerations are generally large and painful, and may be covered by a fibrin membrane. They appear in both keratinized and non-keratinized tissues, and are associated with granulocyte counts of under 800 cells/μl.

•Bacterial infections

During chemotherapy, saprophytic bacteria can become aggressive as a result of the decreased granulocyte presence and increased fragility of the oral mucosa. A number of bacteria, such as Streptococcus viridans, Prevotella, Fusobacterium, Actinobacillus, Actinomycetemcomitans and Actinomyces are associated with infections of the oral cavity in patients receiving chemotherapy (8). Bacterial infections usually manifest locally in the gingival tissue, mucosa and teeth. Necrotizing gingivitis is the most frequent oral manifestation, accompanied in some cases by fever and adenopathies, particularly in patients with previous periodontal conditions. These infections are usually treated administering a combination of penicillins and metronidazole, with subsequent dental treatment (e.g., tartrectomy).

•Fungal infections

The majority of fungal infections of the oral cavity are produced by Candida albicans (4). The most prevalent forms of candidiasis are the pseudomembranous presentation, followed by erythematous candidiasis and angle cheilitis (4,8). Oral infection may give rise to sepsis and can prove fatal if not adequately diagnosed, especially when caused by non-C. albicans species such as Candida tropicalis (8). The diagnosis is based on the clinical appearance of the lesions, the ease with which the necrotic surface of the lesions can be removed by friction, and potassium hydroxide smear preparations, which reveal the presence of the fungus (5). Although prophylactic treatment with antifungal drugs has been questioned, good results have been obtained with such treatment in immune suppressed and/or neutropenic patients (8). In the review of 17 studies published by Lalla et al., the prophylactic administration of fluconazole during cancer therapy was seen to reduce the prevalence of clinically manifest fungal infections, including systemic infections, to 1.9% (8). Topical and systemic antifungal treatment is used for oral candidiasis, combining nystatin (100,000 IU/ml 3-4 times/day) and fluconazole (100 mg/day) or ketoconazole (200 mg/day). In the case of resistance to these drugs, use is made of itraconazole (200-400 mg/day) or amphotericin B, in patients with very extensive and severe infections (20 mg/day) (4,8).

•Viral infections

In most cases, viral infections produced by herpes simplex virus, varicella zoster virus and Epstein-Barr virus are the result of reactivation of a latent virus, while infections produced by cytomegalovirus can be due to reactivation of a latent virus or the action of a recently acquired virus (4,8).

Infection produced by herpes simplex virus (HSV): The incidence of oral lesions produced by recurrent HSV in cancer patients with bone marrow suppression has decreased considerably following the introduction of prophylactic acyclovir (4,8). In patients without antiviral prophylaxis, the oral lesions generally manifest with chemotherapy or chemotherapy-radiotherapy during the most intense immune suppression period. The clinical picture tends to be atypical, with painful ulcerations as a first manifestation. These lesions are crater-shaped, well defined with whitish margins, and are mainly located on the palate and gums (21). The ulcers tend to progress towards mucocutaneous lesions in a short period of time, and are slow in healing. The diagnosis is usually based on the clinical findings, though in some cases viral culture and isolation is recommended in order to confirm the diagnosis and avoid spreading of the lesions (21). Treatment consists of acyclovir via the oral (200-400 mg/3-5 times a day) or intravenous route (5 mg/kg in infusion during one hour every 8-12 hours), for as long as lesions remain (8,22). We can also use oral valacyclovir (500-100 mg twice a day), though the review published by Glenny et al. (22) did not find this form of therapy to be more effective than acyclovir. In the case of resistance to the drug, intravenous foscarnet or cidofovir is an alternative option.

Infection produced by Epstein-Barr virus (EBV): There have been reports of hairy leukoplakia in patients subjected to chemotherapy due to acute myeloid leukemia, acute lymphocytic leukemia and multiple myeloma (8). The infection is clinically characterized by elongated and elevated white lesions located bilaterally at the lateral margins of the tongue. The lesions are not removed by rasping, and produce no symptoms. In general, no treatment is indicated, since the lesions improve as the host immune function recovers, and they do not undergo malignant transformation – though high-dose oral valacyclovir is a safe and effective management option (23). Topical treatments in the form of 25% podophyllin resin either alone or in combination with topical 5% acyclovir and gentian violet have also been found to be safe and effective (8).

Infection produced by varicella-zoster virus (VZV): In contrast to HSV, the orofacial lesions produced by VZV generally manifest several weeks after the interruption of chemotherapy (4). The patients may experience a series of nonspecific prodromic symptoms (pain or dysesthesias), followed by a vesicular eruption along a dermatome innervated in the maxillofacial territory by a trigeminal nerve branch – though in some cases multiple dermatomes can be affected; the lesions may exhibit a more generalized distribution with more extensive skin necrosis; or alternatively there may be extensive hematogenous spread towards muco-cutaneous structures and also internal organs. The pain is described as constant and burning, and the vesicles (blisters) appear both on the skin and on the mucosal membranes, without extending beyond the midline. Depending on the immune depression of the patient, treatment consists of acyclovir (800 mg 5 times a day during 5-7 days via the oral route, or 5-10 mg/kg three times a day during 5 days via the intravenous route), oral famcyclovir or valacyclovir, or intravenous foscarnet (40 mg/kg three times a day) in the case of resistance to the aforementioned drugs. Amitriptyline (25-50 mg/day via oral) or anticonvulsivants (clonazepam, carbamazepine) can be used to treat the pain.

Infection produced by cytomegalovirus (CMV): The lesions produced by CMV consist of multiple nonspecific, pseudomembranous ulcerations covered by a fibrin exudate, with a granulomatous base and irregular margins. At present, gancyclovir is the treatment of choice in cases of acute CMV infection (4).

7.Bleeding tendency

-Etiopathogenesis

Bleeding tendency is secondary to bone marrow suppression caused by chemotherapy or the liver toxicity of certain cytostatic drugs, resulting in alteration of the synthesis of different coagulation factors. Bleeding tendency in the oral cavity usually appears after trauma during chewing in patients with preexisting periodontal disease - especially patients with prior gingivitis and a platelet count of under 20,000 platelets/mm3).

-Clinical characteristics

Clinically, we can observe petechiae, ecchymosis, hematomas or diffuse bleeding in any location of the oral cavity.

-Treatment

Oral rinses with 0.12% chlorhexidine avoid overinfection and can help eliminate remaining blood, though caution is required not to disturb the blood clots, since this may lead to further bleeding (4). The treatment of choice in cases of bleeding consists of vasoconstrictors such as topical norepinephrine, mucoadherent tissue protectors such as cyanoacrylate, and coagulation-favoring drugs such as topical thrombin or hemostatic collagen, which organize and stabilize the blood clots (4). In individuals subjected to chemotherapy who require invasive dental treatment, the hematological condition of the patient must be taken into account, with consultation of the supervising oncologist. In the presence of a platelet count of under 50,000 platelets/mm3, it is advisable to provide invasive dental treatment in the hospital setting, following transfusion assessment.

8.Osteonecrosis of the jaws due to bisphosphonates

-Terminology

Bisphosphonates (BPs) are potent inhibitors of osteoclastic bone reabsorption and have been used for decades for the treatment of osteoporosis, malignant hypercalcemia, solid tumor bone metastases and myeloma (4,24,25). In the year 2003, Marx published a first series of 36 patients with osteonecrosis of the jaws (ONJ) induced by the BPs zolendronate and pamidronate (26). Posteriorly, Ruggiero et al. published a larger series of 63 patients (27). In Spain, Bagán et al., in the year 2005, published a series of 10 patients subjected to chemotherapy with BPs, and who developed osteonecrosis of the jaws (28). With the purpose of exploring the growing problem posed by this new disorder, the American Association of Oral and Maxillofacial Surgeons (AAOMS)(29) in 2007 defined the diagnostic criteria of osteonecrosis of the jaws induced by bisphosphonates. During that same year in Spain, a panel of experts in Oncology, Hematology, Urology, Stomatology / Odontology and Maxillofacial Surgery published a series of recommendations for patients treated with BPs (30).

-Etiopathogenesis

**Figure 1:**
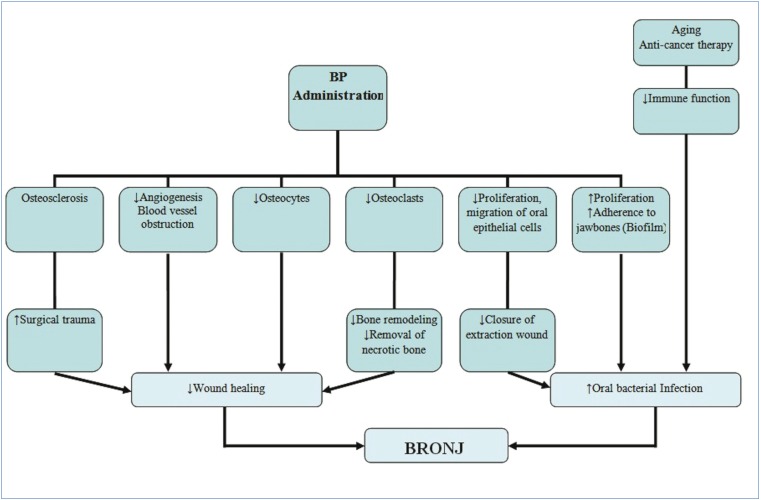
Hypothesis on the mechanism underlying osteonecrosis of the jaws. Adapted from Yoneda et al. (25) // BRONJ: bisphosphonate-related osteonecrosis of the jaw; BP: bisphosphonates.

The etiology of ONJ remains unclear, and although the underlying mechanism of action has not been fully established, a series of factors are believed to be involved. In this context, it has been postulated that BPs produce osteoclast apoptosis, thereby inhibiting bone resorption and bone remodeling, and favoring the creation of areas of bone sequestration (4,24,25,30-33). On the other hand, it has been suggested that inhibition of this homeostatic cycle gives rise to the accumulation of non-vital osteocytes and microfractures in the old bone matrix, thereby facilitating the progression of ONJ (25). Some studies in turn relate ONJ to an increase in bacterial microfilm, favoring bacterial adherence to the surface of the tooth – particularly bacteria belonging to the genus Actinomyces. This could explain why such osteonecrosis only appears in the oral cavity (24,25). On the other hand, BPs may produce blood vessel obstruction within bone, and consequently necrosis of the osteocytes surrounding these vessels. By inhibiting angiogenesis, healing is delayed (25,31-33). In relation to this phenomenon, ONJ has also been observed in patients treated with bevacizumab (25). The inhibition of oral epithelial cell proliferation and migration produced by BPs may cause a delay in post-extraction socket healing. In turn, the accumulation of BPs produces alveolar bone sclerosis, complicating extraction and prolonging the healing time. Lastly, in patients of old age and/or subjected to treatment with chemotherapeutic drugs or corticosteroids, immune function may be altered, thereby increasing the susceptibility to infections (25,31) (Fig. 1).

-Risk factors

The development of ONJ has been associated to a number of general risk factors, such as the type of BP administered, the duration of treatment, the type of neoplasm, the existence of concomitant treatments (chemotherapy, head and neck radiotherapy, corticosteroids, thalidomide or bortezomib), and the presence of other disease conditions (anemia, diabetes, obesity, hypercalcemia and coagulation disorders)(25,32-36). Local risk factors in turn include dentoalveolar surgery, the mandibular location, bone protuberances (torus, mylohyoid crest) and concomitant oral disease (periodontal or dental infections)(4,25,34-36). Regarding hereditary factors, ONJ has been related to polymorphisms of cytochrome P450-2C [CYP2C8] and to the COLIA-1, RANK, MMP-2, OPG, OPN, FPPS and FCEV genes (25,33,35,37). Other contributing cofactors are alcohol, smoking, deficient oral hygiene, obesity and old age (25,33,32,36).

-Incidence

Osteonecrosis of the jaws produced by intravenous BPs is more frequent than ONJ due to oral BPs, with an incidence of 1-12% versus 0.01-0.04%, respectively (24,36,38). In the study published by Ruggiero et al. (34), based on case series, case-control series and cohort studies of patients treated with intravenous BPs up until the year 2006, the cumulative incidence varied between 0.8%-12%. In later studies such as that of Bagán et al. (2009), the suggested incidence was 1-3% (33).

5.Clinical characteristics

Clinically, the onset of ONJ can be nonspecific. The patient may describe discomfort around a tooth, a lack of healing after tooth extraction, or ulceration of the oral mucosa (4,31). As the lesions advance, the patient may develop pain, exposure of necrotic bone, fistulization, purulent secretion, alveolar nerve paresthesia, dental mobility, involvement of the maxillary sinus, and mandibular fracture (31,32,35). In 2006, Ruggiero et al. (39) proposed a staging system for ONJ based on the clinical characteristics of the lesion. In 2009, the AAOMS published a modification of the staging system developed by Ruggiero et al. (34), and posteriorly, in 2012, Bagán et al. (40) proposed a new classification of ONJ with the creation of two new subcategories in stage 2 ( Table 3).

**Table 3:**
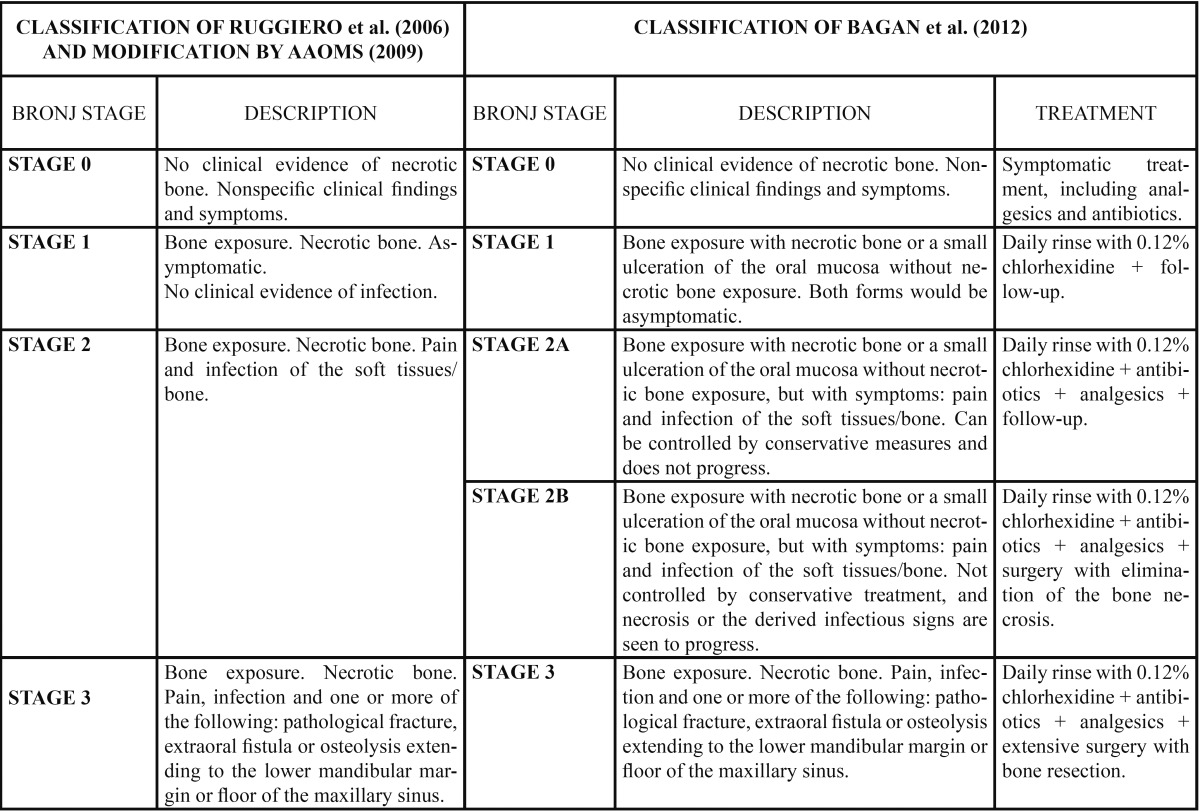
Clinical classification of ONJ developed by Ruggiero (2006), posteriorly modified by the American Association of Oral and Maxillofacial Surgeons (AAOMS, 2009) and the classification of Bagán et al. (2012) (39,34,40).// BRONJ: bisphosphonate-related osteonecrosis of the jaw.

-Complementary tests

A routine panoramic X-ray study is indicated. Computed tomography or magnetic resonance imaging can be used to evaluate the magnitude and extent of necrotic bone, though the latter technique is less specific (30,33). Other complementary tests include culture and an antibiogram of the exposed zone. A biopsy is advised in cases of doubt in differentiating between ONJ and bone metastasis (4,30). The evaluation of serum C-terminal telo-peptide (CTX) is subject to controversy; some studies have found no statistically significant relationship between the CTX levels and the size or number of exposed necrotic bone areas in ONJ (33,35).

-Prevention 

Since the treatment of ONJ is usually unsatisfactory and the condition proves difficult to control, management should focus on the prevention of high risk situations, checking the oral cavity with the purpose of carrying out treatment – especially when of a surgical nature – between 4-6 weeks before the first infusion of BPs (4,25,30,35). If the patient is receiving treatment with BPs, it is advisable to evaluate the oral cavity every 6-12 months (4,30,35), and any required dental treatment should follow a series of measures designed to lessen the risk of ONJ, since some therapies are considered acceptable while others are contraindicated (4,32,34,35).

-Treatment

The treatment of ONJ is controversial, and no effective or fully consensus-based guidelines have been established, though a number of management strategies have been used, such as the interruption of BPs, surgical treatment, the use of hyperbaric oxygen, and the application of ozone, laser surgery, or low-intensity laser therapy (25,30-32,34,35,38). Research is still being conducted on the efficacy of the treatment of ONJ with pentoxy-phylline, α-tocopherol or teriparatide (38). Most authors agree that conservative management of ONJ is the best approach (4,28,35), since mucosal healing can be achieved in at least 23-53% of all patients by adopting less aggressive treatments (33).
